# Characterization of Virulence Properties in the *C. parapsilosis Sensu Lato* Species

**DOI:** 10.1371/journal.pone.0068704

**Published:** 2013-07-09

**Authors:** Tibor Németh, Adél Tóth, Judit Szenzenstein, Péter Horváth, Joshua D. Nosanchuk, Zsuzsanna Grózer, Renáta Tóth, Csaba Papp, Zsuzsanna Hamari, Csaba Vágvölgyi, Attila Gácser

**Affiliations:** 1 Department of Microbiology, University of Szeged, Szeged, Hungary; 2 Department of Microbiology and Immunology, Albert Einstein College of Medicine, New York, New York, United States of America; Instituto de Salud Carlos III, Spain

## Abstract

The *C. parapsilosis sensu lato* group involves three closely related species, *C. parapsilosis sensu stricto*, *C* *. orthopsilosis* and *C* *. metapsilosis* . Although their overall clinical importance is dramatically increasing, there are few studies regarding the virulence properties of the species of the *psilosis* complex. In this study, we tested 63 *C. parapsilosis sensu stricto*, 12 *C* *. metapsilosis* and 18 *C* *. orthopsilosis* isolates for the ability to produce extracellular proteases, secrete lipases and form pseudohyphae. Significant differences were noted between species, with the *C* *. metapsilosis* strains failing to secrete lipase or to produce pseudohyphae. Nine different clinical isolates each of *C. parapsilosis sensu stricto*, *C* *. orthopsilosis* and *C* *. metapsilosis* were co-cultured with immortalized murine or primary human macrophages. *C. parapsilosis sensu stricto* isolates showed a significantly higher resistance to killing by primary human macrophages compared to *C* *. orthopsilosis* and *C* *. metapsilosis* isolates. In contrast, the killing of isolates by J774.2 mouse macrophages did not differ significantly between species. However, *C. parapsilosis sensu stricto* isolates induced the most damage to murine and human macrophages, and *C* *. metapsilosis* strains were the least toxic. Furthermore, strains that produced lipase or pseudohyphae were most resistant to macrophage-mediated killing and produced the most cellular damage. Finally, we used 9 isolates of each of the *C. parapsilosis sensus lato* species to examine their impact on the survival of *Galleria* *mellonella* larvae. The mortality rate of *G* *. mellonella* larvae infected with *C* *. metapsilosis* isolates was significantly lower than those infected with *C. parapsilosis sensu stricto* or *C* *. orthopsilosis* strains. Taken together, our findings demonstrate that *C* *. metapsilosis* is indeed the least virulent member of the *psilosis* group, and also highlight the importance of pseudohyphae and secreted lipases during fungal-host interactions.

## Introduction

Members of *Candida parapsilosis sensu lato* are emerging opportunistic pathogens especially notable for causing nosocomial infections worldwide [1]. The *C. parapsilosis sensu lato* group involves three closely related species, *C. parapsilosis sensu stricto*, *C* *. orthopsilosis* and *C* *. metapsilosis* . Before 2005, isolates of all three species were considered as *C. parapsilosis*; however, molecular differences subsequently led to the separation into the discrete species [2]. Although closely related, the members of *C. parapsilosis* complex differ markedly from each other in regard to their clinical prevalence and, purportedly, in virulence. *In vitro* infection models have suggested that *C* *. metapsilosis* is the least virulent species of the *psilosis* group [3], [4], which is in line with its low clinical prevalence compared to *C. parapsilosis sensu stricto* and *C* *. orthopsilosis* [5], [6]. Furthermore, according to a recent study, isolates of *C* *. metapsilosis* adhere less avidly to human buccal epithelial cells *in vitro* and are less virulent in a murine model of experimental vaginal candidiasis in comparison to *C. parapsilosis sensu stricto* and *C* *. orthopsilosis* [7].

As the *C. parapsilosis sensu lato* group is phylogenetically closely related yet differing in virulence, these species represent an exquisite system for the study of the molecular mechanisms that have enabled opportunistic microorganisms to adapt to the human host.

Despite enormous progress in deciphering the pathogenesis of certain fungi, such as *Candida albicans*, little is known about the genetic basis of fungal virulence traits that enable *C. parapsilosis sensu lato* species to cause disease. Notably, secretion of hydrolytic enzymes such as aspartyl proteinases and lipases, and the ability of morphogenetic switch between yeast and mycelial growth have been clearly associated with *C. albicans* virulence [8], [9] [10],. In addition to simply assisting in the digestion of molecules for nutrient acquisition, extracellular proteinases of pathogenic fungi play specialized roles during infections. They may digest or distort host cell membranes to facilitate adhesion and tissue invasion, or damage cells and molecules of the host immune system to avoid or resist antimicrobial attack [11]. Our group has recently shown that secreted aspartyl proteinases play an important role in the virulence of *C. parapsilosis sensu stricto* [12]. Microbial extracellular lipases are involved in the hydrolysis of different lipid molecules for nutrient acquisition, adhesion to host cells and the synthesis or degradation of specific inflammatory mediators, thereby influencing the immune response of the host [9]. Secreted lipase of *C. parapsilosis* was clearly associated with virulence when secreted lipase null mutants were found to poorly form biofilm and they had increased susceptibility to *in vitro* killing by macrophages and reduced virulence in reconstituted human oral epithelium or murine intraperitoneal challenge [13]. Morphogenetic switching of *C. albicans* from yeast into filamentous phase is regarded as a major virulence factor that enables evasion of phagocytosis [10]. Although the members of the *C. parapsilosis sensu lato* group do not develop true hyphae, they are able to produce elongated cells called pseudohyphae and this morphotype is a potential virulence factor.

In this study, we examined three important virulence factors (pseudohypha formation, extracellular lipase and proteinase production) of different *C. parapsilosis sensu stricto,* *C* *. orthopsilosis* and *C* *. metapsilosis* isolates. Additionally, we used both conventional and non-conventional models to further explore the virulence properties of these species. We examined interactions and outcomes of murine and human macrophages with different isolates of the *C. parapsilosis sensu lato* species. Additionally, we utilized *Galleria* *mellonella* larvae as an *in vivo* model system [14], [15] to compare the virulence of these species. To our knowledge, this is the most extensive study to date to explore and compare the virulence properties of the members of *C. parapsilosis* species complex.

## Materials and Methods

### Strains and growth conditions

*Candida* strains used in this study are listed in Table S1. PCR products of the ITS region of the strains were sequenced to confirm their species-level taxonomic classification using ITS1 (TCCGTAGGTGAACCTGCGG) and ITS4 (TCCTCCGCTTATTGATATGC) primers as described by Kocsubé et al [16].

*Candida* strains were maintained on YPD plates (1% yeast extract, 2% bactopepton, 2% glucose, 2.5% agar) at 4 °C. Prior to the experiments, strains were grown overnight in liquid YPD medium (1% yeast extract, 2% bactopepton, 2% glucose) at 30 °C in a shaker incubator. Cells were harvested by centrifugation, washed twice with PBS (phosphate-buffered saline; 137 mM NaCl, 2.7 mM KCl, 10 mM Na_2_HPO_4_, 2 mM KH_2_PO_4_; pH 7.4), counted in a Bürker-chamber and adjusted to the proper concentration detailed for each experiment.

### Secreted lipase assay

Lipolytic activities were examined by growing *Candida* colonies on YNB plates (Yeast Nitrogen Base, 2.5% agar) supplemented with 2% commercially available olive oil and 1 mM rhodamine B (Sigma-Aldrich) [17]. Aliquots of 10 µL of each of the different *Candida* isolate suspensions containing 10^6^ cells/mL were inoculated in duplicate onto rhodamine B olive oil agar plates and incubated at 30 °C for 7 days. In the presence of secreted lipase, substrate hydrolysis causes the formation of orange fluorescent halos around colonies visible upon UV irradiation and colonies then acquire a dark pinkish color change. Isolates were considered lipase positive when a pink halo was detectable around the colonies.

### Secreted proteinase assay

*C. parapsilosis sensu lato* isolates were analyzed for secreted proteolytic activity on solid medium containing BSA (Bovine Serum Albumin, Sigma-Aldrich) as the sole nitrogen source. Aliquots of 10 µL of each of the different *Candida* isolate suspensions containing 10^6^ cells/mL were inoculated in duplicate onto YCB-BSA agar plates (1.17% yeast carbon base, 0.01% yeast extract, 0.2% BSA; pH 5.0) and incubated at 30 °C for 7 days. Proteolysis was determined by amido black (Sigma-Aldrich) staining of the BSA present in the medium as described by Ruchel and colleagues [18]. Proteinase activity was considered to be absent when no clarification of the medium around the colony was visible. When the proteolytic halo was visible we considered the isolate protease positive.

### Pseudohypha production

To examine pseudohypha formation, individual wells of 24-well plastic cell culture plates containing 1 mL DMEM medium (Lonza) supplemented with 10% FBS (Fetal Bovine Serum; Lonza) and 1% 100x Penicillin-Streptomycin solution (Sigma-Aldrich) were inoculated with 50 µL of suspensions containing 6x10^6^ cells/mL of the different *C. parapsilosis sensu lato* isolates. Examination for pseudohypha formation was performed after 24 hours using light microscopy. Strains were considered positive for pseudohypha formation when chains of budding cells could be seen in the culture.

### Murine cell line

The murine macrophage-like cell line J774.2 (Invitrogen) was cultured in DMEM medium (Lonza) supplemented with 10% heat-inactivated FBS (Lonza) and 1% 100x Penicillin-Streptomycin solution at 37 °C, 5% CO_2_ and 100% relative humidity. All J774.2 macrophage experiments were performed in this medium.

### Human PBMC isolation and macrophage differentiation

Human peripheral blood mononuclear cells (PBMCs) were isolated from buffy coats of healthy donors by Ficoll Paque PLUS (GE Healthcare) density gradient centrifugation as described previously [19]. Experiments were performed according to the institutional regulation of the independent ethics committee of the University of Szeged. PBMCs were washed with PBS and treated with ACK lysis buffer (150 mM NH_4_Cl, 10 mM KHCO_3_, 0.1 mM Na _2_EDTA) in order to eliminate erythrocytes. Isolated PBMCs were suspended in RPMI medium (Lonza) supplemented with 10% heat-inactivated FBS or human serum (Lonza) and 1% 100x Penicillin-Streptomycin solution (Sigma-Aldrich), and plated on 6- or 12-well cell culture plates to isolate monocytes by plastic adherence. After 2 hours of incubation (37 °C, 5% CO_2_, 100% relative humidity), floating cells were removed, and the attached monocytes were gently washed with PBS. The isolated cells were cultured for 7 days in the presence of 10% heat-inactivated FBS or human serum to enable macrophage differentiation. Experiments with human macrophages were performed in RPMI medium supplemented with 10% heat-inactivated FBS or human serum and 1% 100x Penicillin-Streptomycin solution.

### Measurement of LDH (lactate dehydrogenase) *activity*

LDH activity in cell culture supernatants was measured using the Cytotoxicity Detection Kit (LDH; Roche) according to the manufacturer’s instructions. Macrophages were stimulated with *C. parapsilosis sensu stricto*, *C* *. orthopsilosis* or *C* *. metapsilosis* cells at a ratio of 1:5 for 24 or 48 hours, or left unstimulated (negative control). During analysis, the LDH activity measured in cultures containing yeast cells alone was subtracted from the values measured in stimulated samples.

### Killing assay

Murine (J774.2) or primary human macrophages were co-incubated in plastic cell culture plates with different *C. parapsilosis sensu lato* isolates at an effector/target ratio of 1:5. As a control, the same number of yeast cells were incubated in the appropriate cell culture medium without macrophages. After 3 hours of incubation, macrophages were lysed by forcibly pulling the culture through a 26-gauge needle 5 times. The lysates were then serially diluted, plated on YPD agar plates and incubated at 30 °C. After 2 days, the number of CFUs (colony forming units) was determined and multiplied by the dilution factor to calculate the original cell number. The killing efficiency was calculated as follows: [(number of live *Candida* cells in control wells – number of live *Candida* cells in co-cultures) / number of live *Candida* cells in control wells] x 100.

### Phagocytosis assay (quantitative imaging)

For the analysis of phagocytosis by quantitative imaging, yeast cells were labeled with the fluorescent dye Alexa Fluor 647 carboxylic acid, succinimidyl ester (Invitrogen). Briefly, 11 µL Na _2_CO_3_ (1 M, pH 10) and 2 µL Alexa Fluor 647 (1 mg/mL, in DMSO) was added to 100 µL of the *Candida* cell suspensions containing 10^9^ cells/mL in PBS, and incubated in the dark for 30 min at room temperature. Afterwards, cell suspensions were washed four times with PBS, counted in a Bürker-chamber and adjusted to the proper concentration. Murine J774.2 macrophages were co-cultured with the labeled *Candida* strains in 12-well plastic cell culture plates at a ratio of 1:5 for two hours to allow phagocytosis. After the incubation period, the cell culture medium was removed, and macrophages were washed extensively with PBS in order to eliminate non-phagocytosed *Candida* cells. Macrophages were then gently suspended to a single cell suspension by pipetting, harvested by centrifugation, resuspended in 50 µL FACS buffer (0.5% FBS in PBS) and measured on a FlowSight instrument (Amnis). Data were analysed using the IDEAS Software (Amnis).

### *Galleria* *mellonella* survival assay

Infection of *G* *. mellonella* larvae (Mous Livebait R.J., The Netherlands) was performed as described previously [21,22]. Briefly, larvae were inoculated with 10 µL of a *Candida* suspension prepared in PBS containing 6x10^6^ yeast cells by an injection in the last left pro-leg, using a 26 gauge needle with Hamilton syringes. After injection, caterpillars were incubated at 25 °C, and the number of dead larvae was noted daily. In each experiment, a group of caterpillars were left without any manipulation (untreated control), and another group of caterpillars was inoculated with PBS only.

### Statistical analysis

Statistical significance was determined by an appropriate nonparametric test (Kruskal-Wallis or Mann-Whitney test), using the GraphPad Prism v 5.0 software. In some cases, significance could not be determined by GraphPad Prism, therefore, the R 2.15.2 software was used (exact Wilcoxon rank sum test, see figure legends). Differences between groups were considered statistically significant at P<0.05.

## Results

### Extracellular lipase- and protease-producing and pseudohypha-forming ability of *C. parapsilosis sensu lato* isolates

**Figure 1:**
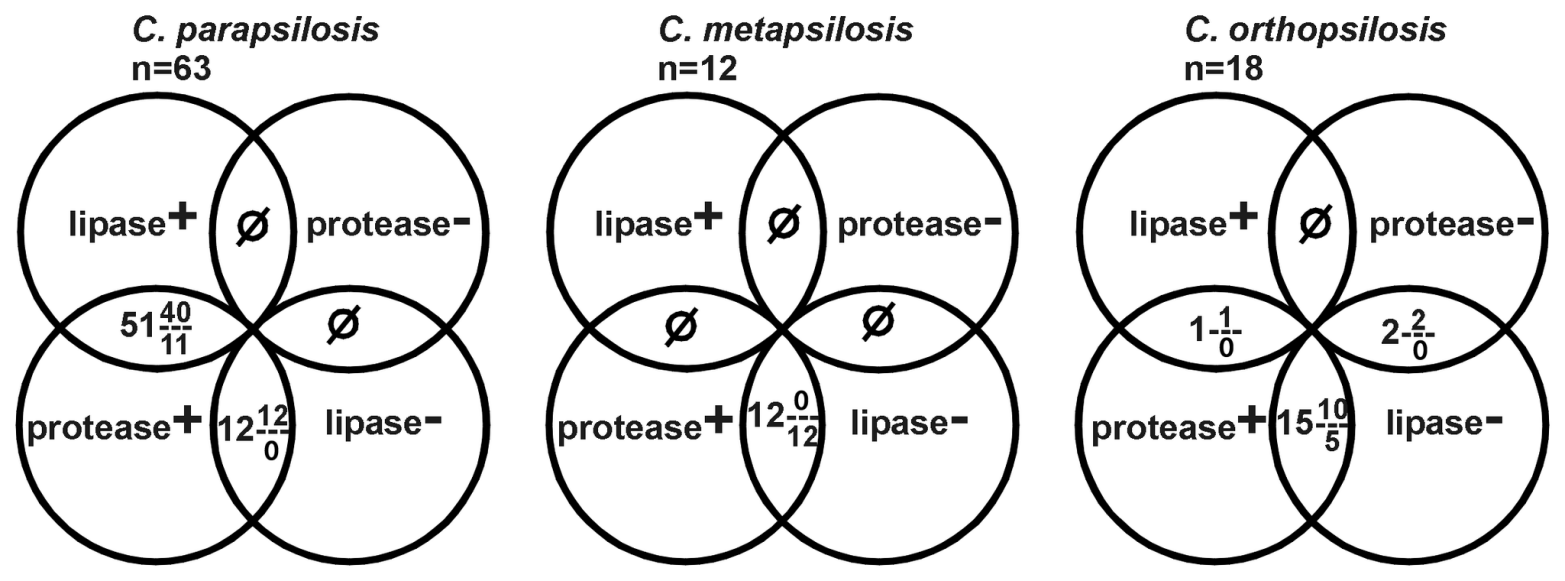
Diagrammatic presentation of the occurrence of lipase-, protease- and pseudohyphae-producer strains in the *C. parapsilosis sensu lato* complex. n: number of isolates, upper number in fraction: number of pseudohypha-producer strains, lower number in fraction: number of pseudohypha negative strains.

In the first set of experiments, 63 *C. parapsilosis sensu stricto*, 12 *C* *. metapsilosis* and 18 *C* *. orthopsilosis* isolates were monitored for three biological attributes known to play a role in virulence: extracellular lipase and protease activity as well as the ability to form pseudohyphae (Table S1.). We found that all of the 63 *C. parapsilosis sensu stricto* isolates possessed secreted protease activity, and 51 of them were positive for lipase production. Pseudohypha production varied in the lipase/protease positive group with 40 of the 51 forming pseudohyphae. All of the lipase negative protease positive *C. parapsilosis sensu stricto* isolates produced pseudohyphae. The 12 *C* *. metapsilosis* isolates were protease positive, lipase negative and lacked the ability of pseudohypha production. For the 18 *C* *. orthopsilosis* isolates, 15 were protease positive and lipase negative, and 10 from this group formed pseudohyphae. One *C* *. orthopsilosis* isolate secreted both lipase and protease as well as formed pseudohyphae, and 2 isolates secreted neither enzyme yet produced pseudohyphae. Figure 1 summarizes the occurrence of lipase-, protease- and pseudohypha-producing ability in different combinations among *C. parapsilosis sensu lato* isolates.

### Interactions of *C. parapsilosis sensu lato* species with J774.2 murine macrophages: fungal killing, host cell damage and phagocytosis

**Figure 2:**
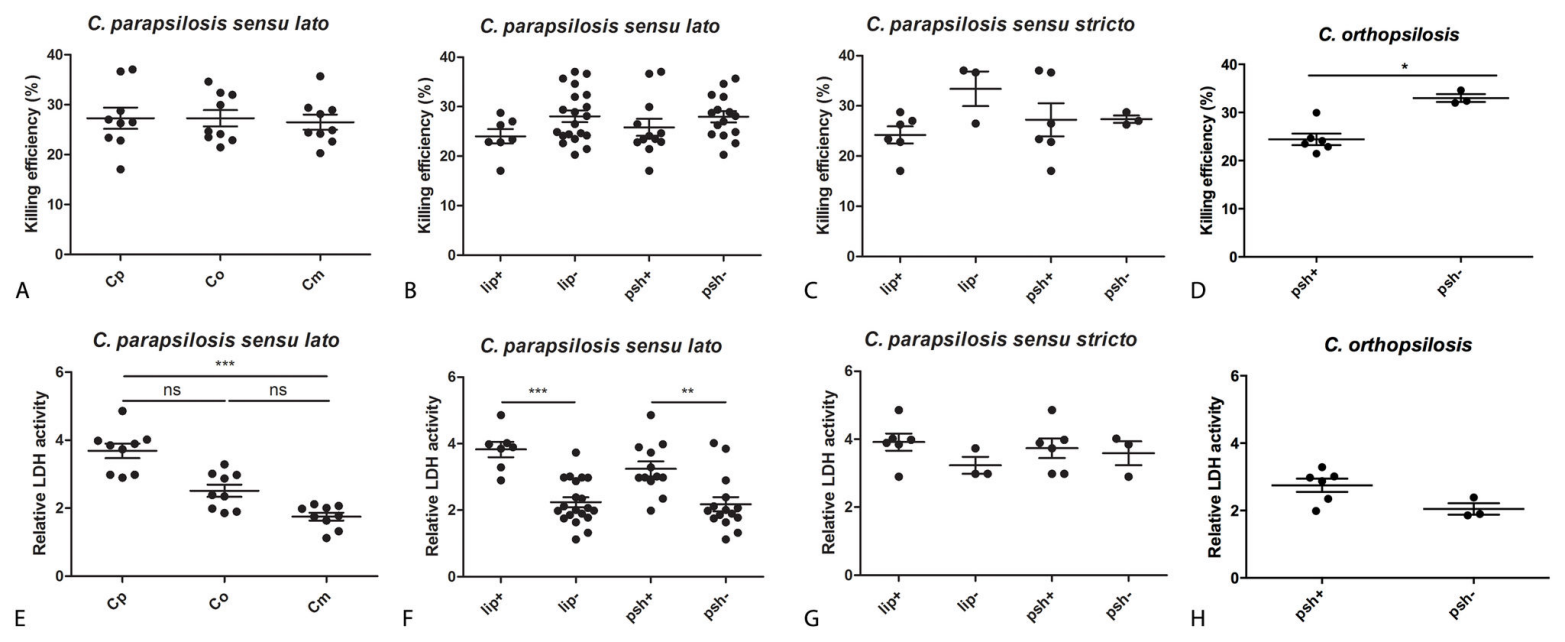
Interactions of *C. parapsilosis sensu lato* isolates (see Table S1.) with J774.2 murine macrophages. (A) Killing efficiency of *C. parapsilosis sensu lato* species determined by CFU determinations (Cp, *C. parapsilosis sensu stricto*; Co, *C* *. orthopsilosis* ; Cm, *C* *. metapsilosis* ), (B) killing efficiency of lipase producer vs. non-producer and pseudohypha positive vs. negative strains in the *C. parapsilosis sensu lato* group [lip+, lipase positive (regardless of pseudohypha production); lip-, lipase negative; psh+, pseudohyphae positive (regardless of lipase production); psh-, pseudohyphae negative], (C) killing efficiency of lipase or pseudohypha positive vs. negative isolates of *C. parapsilosis sensu stricto*, (D) killing efficiency of lipase or pseudohypha producer vs. non-producer strains of *C* *. orthopsilosis* , (E) host-cell damaging capacity of *C. parapsilosis sensu lato* species based on the release of LDH (lactate dehydrogenase), (F) host-cell damaging capacity of lipase or pseudohypha producer vs. non-producer strains in the *C. parapsilosis sensu lato* group, (G) host-cell damaging capacity of lipase or pseudohypha positive vs. negative isolates of *C. parapsilosis sensu stricto*, (H) host-cell damaging capacity of lipase or pseudohypha producer vs. non-producer strains of *C* *. orthopsilosis* , C.p., *C. parapsilosis sensu stricto*; C.o., *C* *. orthopsilosis* ; C.m., *C* *. metapsilosis* ; cont, control (macrophages without fungal cells). Data points on graphs represent individual strains. Experiments were performed in triplicates. Data were analyzed by the Kruskal-Wallis test (A, E) or the Mann-Whitney test (B, C, D, F, G, H). * p<0.05, ** p<0.01, *** p<0.001.

J774.2 murine macrophage-like cells were used to examine the resistance of *C. parapsilosis sensu lato* species to macrophage-mediated killing. Nine isolates each of *C. parapsilosis sensu stricto,* *C* *. orthopsilosis* and *C* *. metapsilosis* were chosen to include, if possible, isolates with the presence or absence of enzymatic and morphological factors, and their survival was examined after co-culturing them for 3 hours with J774.2 cells. CFU determinations revealed that murine macrophages eliminated the isolates of different *C. parapsilosis sensu lato* species with similar efficiency (mean ± s.d., *C. parapsilosis sensu stricto*: 27.28 ± 6.39%, *C* *. orthopsilosis* : 27.28 ± 4.93%, *C* *. metapsilosis* : 26.50 ± 4.57%, Figure 2A). Furthermore, we found that the killing of extracellular lipase- or pseudohypha-producers was not significantly different from that of lipase or pseudohyphae negative isolates, respectively, in the *C. parapsilosis sensu lato* group (Figure 2B). However, J774.2 macrophages killed the pseudohypha positive strains of *C* *. orthopsilosis* with lower efficiency compared to the pseudohypha negative isolates (mean ± s.d., 24.42 ± 2.93% and 33.00 ± 1.43%, respectively, p<0.05) (Figure 2D). There was also a trend toward less killing of the lipase positive strains of *C. parapsilosis sensu stricto* compared to the lipase negative isolates (24.22 ± 4.17 vs. 33.40 ± 5.99%, respectively), although this difference was not statistically significant (Figure 2C). Since all of the tested *C. parapsilosis sensu lato* isolates were positive for secreted protease production except for two *C* *. orthopsilosis* strains, the contribution of this ability to virulence could not be examined. We also analyzed the *Candida* -induced damage of host cells by measuring the activity of LDH released from dead macrophages. After 24 hours, *C. parapsilosis sensu stricto* isolates caused a significantly higher (mean ± s.d., 3.69 ± 0.64 relative activity) LDH release from J774.2 macrophages than *C* *. metapsilosis* (1.75 ± 0.35 relative activity, p<0.01) (Figure 2E). Although the difference between the host-cell damaging capacity of *C* *. orthopsilosis* versus *C. parapsilosis sensu stricto* and *C* *. orthopsilosis* versus *C* *. metapsilosis* isolates was not statistically significant, a trend could be seen showing LDH activity for *C. parapsilosis sensu stricto* was greater than that for *C* *. orthopsilosis* , which was greater than for *C* *. metapsilosis* . Additionally, our data showed that the lipase negative members of *C. parapsilosis sensu lato* induced significantly lower LDH release from macrophages compared to lipase-producer strains (mean ± s.d., 2.24 ± 0.66 vs. 3.83 ± 0.62 relative LDH activity, respectively, p<0.001, Figure 2F). The tendency of increased LDH-release from macrophages upon infection with lipase positive strains could be also seen in the *C. parapsilosis sensu stricto* group (Figure 2G). The pseudohypha negative isolates of the *C. parapsilosis sensu lato* caused lower damage to host cells compared to pseudohypha-producers (2.18 ± 0.82 and 3.24 ± 0.77 relative LDH activity, respectively, p<0.01, Figure 2F). Similarly, there was a trend toward less damage with pseudohypha negative strains of *C* *. orthopsilosis* compared to pseudohypha positive isolates, but this trend was absent in the *C. parapsilosis sensu stricto* group (Figure 2G and H).

**Figure 3:**
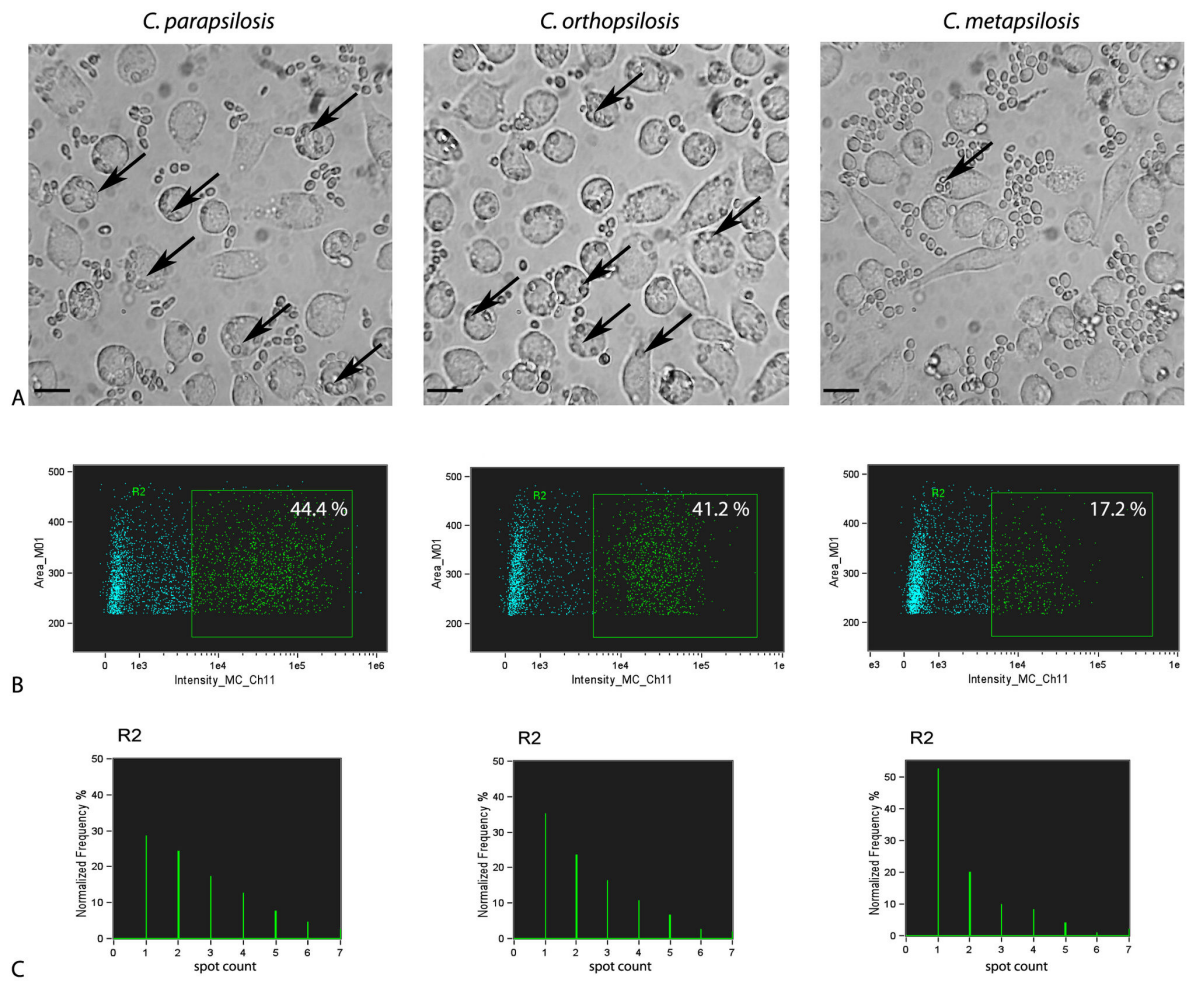
Phagocytosis of one representative isolate each of *C. parapsilosis sensu stricto*, *C* *. orthopsilosis* and *C* *. metapsilosis* by J774.2 macrophages. (A) Light microscopic picures of J774.2 macrophages phagocytosing *C. parapsilosis sensu lato* species, (B) phagocytosis of *C. parapsilosis sensu stricto*, *C* *. orthopsilosis* and *C* *. metapsilosis* by J774.2 macrophages assessed by quantitative imaging, R2: phagocytosing macrophage population discriminated by the presence of red fluorescence due to ingestion of Alexa Fluor 647-labeled yeast cells, (C) numbers of ingested yeast cell/macrophage in the R2 population in case of *C. parapsilosis sensu stricto*, *C* *. orthopsilosis* and *C* *. metapsilosis* determined by the spot-counting feature of IDEAS software.

After the finding that J774.2 macrophages are able to kill all three species of the *C. parapsilosis sensu lato* group with similar efficiency, we examined whether there are differences in the intensity of phagocytosis induced by different species of the *psilosis* group. The phagocytotic activity of J774.2 macrophages against a representative isolate of each of the different species was examined by quantitative imaging. The FlowSight instrument used in these experiments unifies the advances of a flow cytometer and a fluorescent microscope, and has proven very useful for the examination of phagocytosis. Interestingly, we found that after 2 hours of incubation, the ingestion of fungal cells by J774.2 macrophages was considerably lower for *C* *. metapsilosis* (17.2%), compared to *C. parapsilosis sensu stricto* (44.4%) and *C* *. orthopsilosis* (41.2%, Figure 3B). The number of engulfed yeast cells by one macrophage also differed between the three species. In assays with *C* *. metapsilosis* , a single yeast cell was present in over 50% of macrophages containing *Candida* , whereas significantly more macrophages contained multiple *C. parapsilosis sensu stricto* or *C* *. orthopsilosis* (Figure 3C).

Taken together, these data show that although all three species of the *C. parapsilosis sensu lato* group exhibit similar resistance to J774.2 macrophage killing, they differ significantly in their host-cell damaging capacity.

### Killing and host-cell damaging capacity of *C. parapsilosis sensu lato* species assessed by interactions with human peripheral blood mononuclear cell-derived macrophages

**Figure 4:**
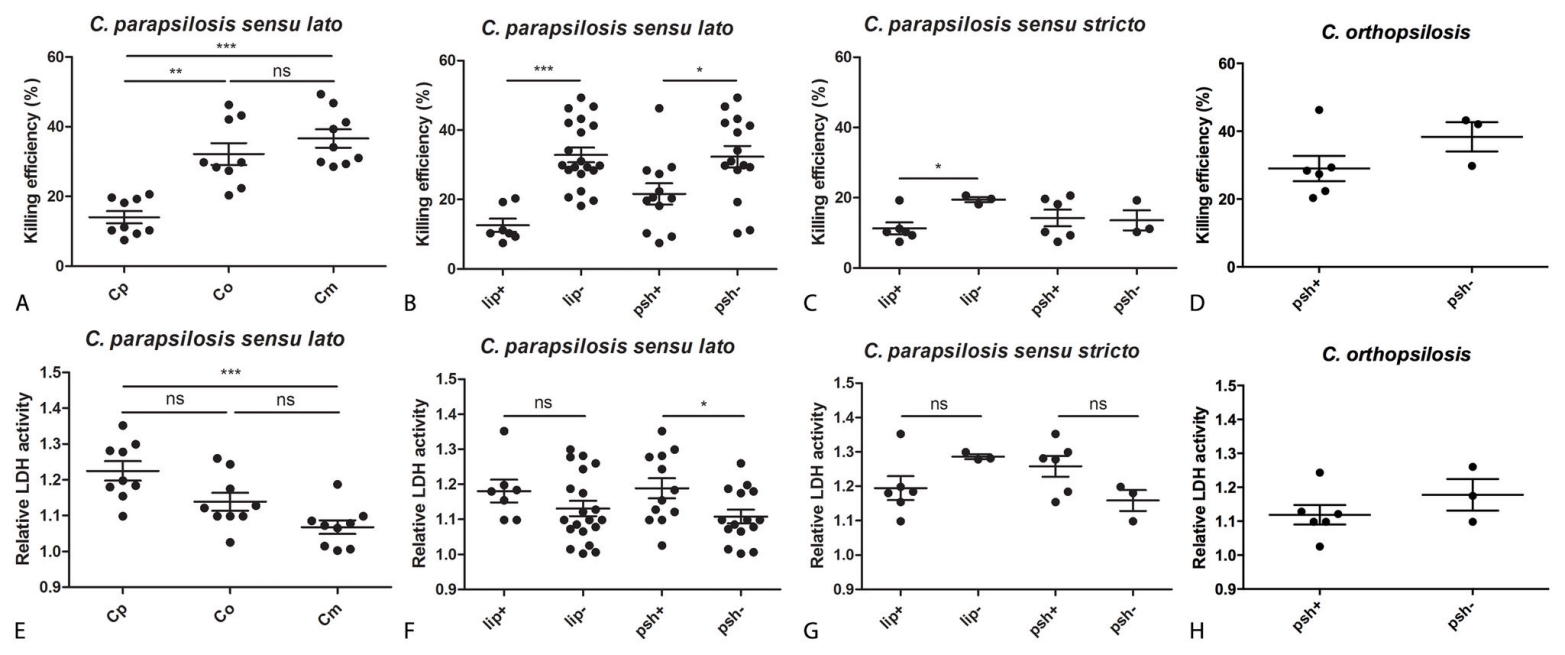
Interactions of different *C. parapsilosis sensu lato* isolates (see Table S1.) with primary human monocyte-derived macrophages. (A) Killing efficiency of *C. parapsilosis sensu lato* species based on CFU-determinations (Cp, *C. parapsilosis sensu stricto*; Co, *C* *. orthopsilosis* ; Cm, *C* *. metapsilosis* ), (B) killing efficiency of lipase producer vs. non-producer and pseudohypha positive vs. negative strains in the *C. parapsilosis sensu lato* group [lip+, lipase positive (regardless of pseudohypha production); lip-, lipase negative; psh+, pseudohyphae positive (regardless of lipase production); psh-, pseudohyphae negative], (C) killing efficiency of lipase or pseudohyphae positive vs. negative isolates of *C. parapsilosis sensu stricto*, (D) killing efficiency of lipase or pseudohyphae producer vs. non-producer strains of *C* *. orthopsilosis* , (E) host-cell damaging capacity of *C. parapsilosis sensu lato* species based on the release of LDH (lactate dehydrogenase), (F) host-cell damaging capacity of lipase or pseudohyphae producer vs. non-producer strains in the *C. parapsilosis sensu lato* group, (G) host-cell damaging capacity of lipase or pseudohyphae positive vs. negative isolates of *C. parapsilosis sensu stricto*, (H) host-cell damaging capacity of lipase or pseudohyphae producer vs. non-producer strains of *C* *. orthopsilosis* . Cp, *C. parapsilosis sensu stricto*; Co, *C* *. orthopsilosis* ; Cm, *C* *. metapsilosis* . Data points on graphs represent individual strains. Experiments were performed in triplicates. Data were analyzed by the Kruskal-Wallis test (A, E), the Mann-Whitney test (B, D, F, G, H) or the Wilcoxon rank sum test (C). * p<0.05, ** p<0.01, *** p<0.001.

To further explore the virulence of the three *C. parapsilosis sensu lato* species, we examined their killing and host cell-damaging capacity using primary human PBMC-derived macrophages. Firstly, we verified that all three *psilosis* species could be efficiently phagocytosed and killed by primary human macrophages using fluorescent microscopy following acridine orange/ crystal violet staining as described by Pruzanski and Saito [20] (data not shown). Afterwards, we assessed the killing of nine isolate each of *C. parapsilosis sensu stricto*, *C* *. orthopsilosis* and *C* *. metapsilosis* by the conventional CFU-counting method. We found that *C. parapsilosis sensu stricto* isolates were killed less efficiently (mean ± s.d., 14.02 ± 5.26%) by human macrophages compared to both *C* *. orthopsilosis* (32.17 ± 9.39%) and *C* *. metapsilosis* (36.61 ± 7.88%) strains (p<0.01, Figure 4.A). Although the killing of *C* *. metapsilosis* isolates was greater than that of *C* *. orthopsilosis* strains, the differences were not significant (p>0.05). Lipase or pseudohyphae positive members of *C. parapsilosis sensu lato* were killed with significantly lower efficiency compared to lipase or pseudohyphae negative isolates (mean ± s.d. killing efficiency, lipase producers vs. non-producers, 12.58 ± 5.07% and 32.83 ± 9.51%, respectively, p<0.001; pseudohyphae positives vs. negatives, 21.62 ± 10.64% and 32.35 ± 11.92%, respectively, p<0.05, Figure 4B). The difference in the killing of lipase producer and non-producer isolates could also be seen in the *C. parapsilosis sensu stricto* group (mean ± s.d. killing efficiency, lipase producers vs. non-producers, 11.29± 4.097% and 19.48± 1.225%, respectively, p<0.05 Figure 4C). When analyzing the host cell-damaging capacity of *C. parapsilosis sensu lato* species, we found that the isolates of *C. parapsilosis sensu stricto* caused the highest LDH-release from human macrophages (mean ± s.d., 1.23 ± 0.08 relative activity), followed by the strains of *C* *. orthopsilosis* (1.14 ± 0.08 relative activity) and then *C* *. metapsilosis* (1.07 ± 0.06 relative activity, Figure 4E). *C. parapsilosis sensu stricto* isolates caused significantly higher damage to human macrophages compared to *C* *. metapsilosis* strains (p<0.001). Although *C. parapsilosis sensu stricto* isolates appeared to cause higher damage to host cells compared to *C* *. orthopsilosis* strains as well, this difference was not statistically significant (p>0.05). Furthermore, we found that pseudohypha positive isolates of *C. parapsilosis sensu lato* induced slightly higher LDH release from macrophages in comparison to negative strains (mean ± s.d., 1.19 ± 0.10 and 1.11 ± 0.08 relative LDH activity, p<0.05, Figure 4F). There was a trend toward greater LDH release of macrophages infected with lipase positive *C. parapsilosis sensu lato* isolates compared to the lipase negative strains (mean ± s.d., 1.18 ± 0.09 and 1.13 ± 0.10 relative LDH activity, respectively Figure 4F). Similarly, there was a trend toward greater damage from pseudohyphae positive *C. parapsilosis sensu stricto* strains (Figure 4G). No significant differences were found in host cell toxicity caused by *C* *. orthopsilosis* pseudohyohae positive and negative strains (Figure 4H).

### Comparison of the virulence of *C. parapsilosis sensu lato* species using the invertebrate model *G mellonella*

**Figure 5:**
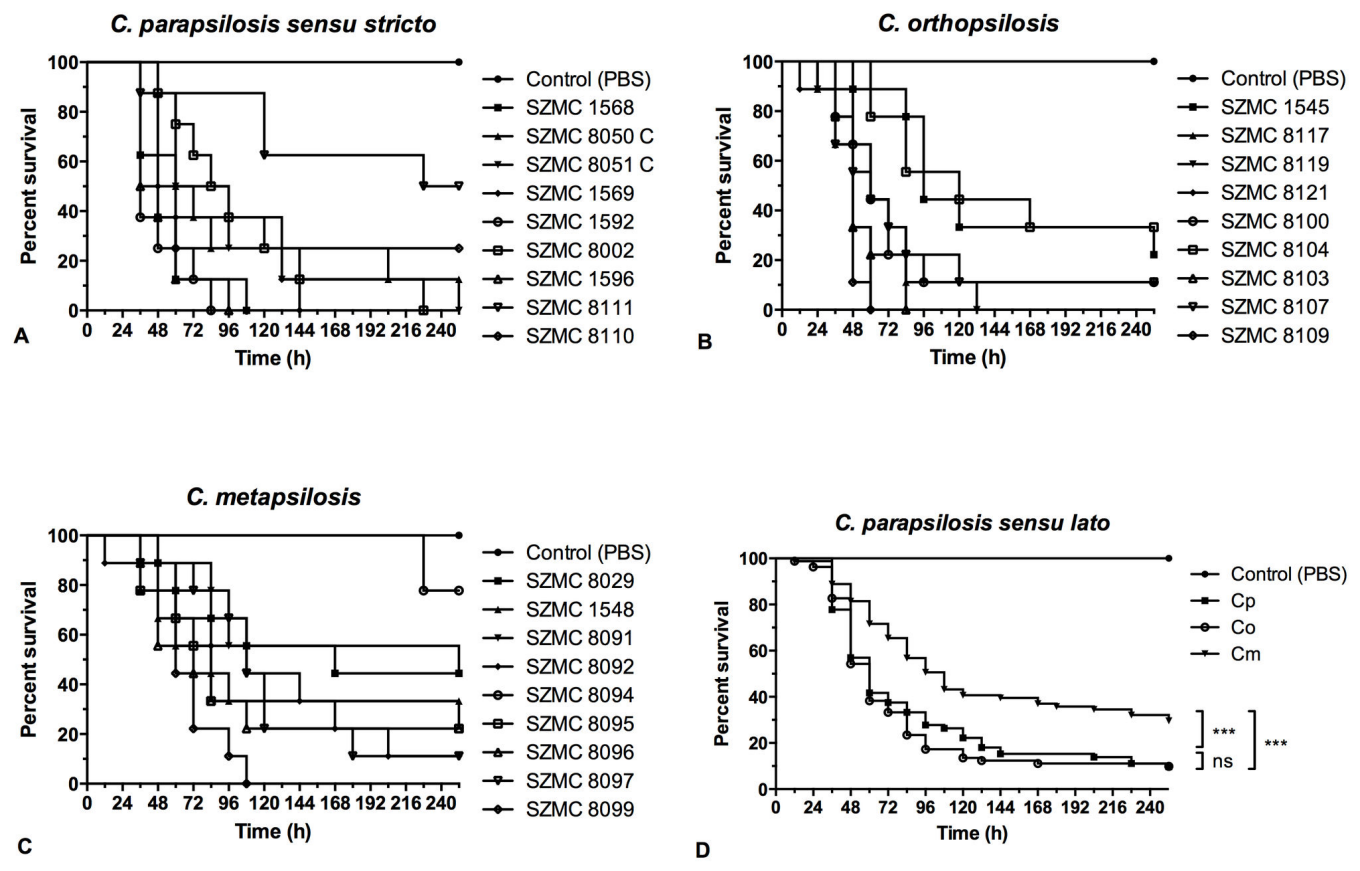
Survival of *Galleria* *mellonella* larvae infected with different isolates of (A) *C. parapsilosis sensu stricto*, (B) *C* *. orthopsilosis* and (C) *C* *. metapsilosis* . For *C. parapsilosis sensu stricto* isolates, each survival group contained 8 larvae, whereas 9 larvae per group were used to assess survival after *C* *. orthopsilosis* or *C* *. metapsilosis* infection. (D) Summarized survival curve of nine *C. parapsilosis sensu lato*; Cp, *C. parapsilosis sensu stricto*; Co, *C* *. orthopsilosis* ; and Cm, *C* *. metapsilosis* isolates. ns, not significant; * p<0.05, ** p<0.01, *** p<0.001 by the log-rank (Mantel-Cox) test.

In all experiment, 8 or 9 larvae were injected with 6x10^6^ yeast cells and their survival was followed for 10 days. In total, 9 different isolates each of *C. parapsilosis sensu stricto*, *C* *. orthopsilosis* and *C* *. metapsilosis* were used. We found that the survival of larvae changed significantly upon infection with any of the three species (*C. parapsilosis sensu stricto* and *C* *. orthopsilosis* , p<0.0001; *C* *. metapsilosis* , p<0.01, as determined by the Log-rank (Mantel-Cox) test), compared to PBS-treatment. Furthermore, our results show that the mortality rate of *G* *. mellonella* larvae infected with *C* *. metapsilosis* isolates was significantly lower than those infected with *C. parapsilosis sensu stricto* or *C* *. orthopsilosis* strains (p<0.001 and p<0.0001, respectively, according to the Log-rank (Mantel-Cox) test) Figure 5A–D. The median survival time of a composite of all *C* *. metapsilosis* -infected larvae was 108 hours, whereas that of *C. parapsilosis sensu stricto*- or *C* *. orthopsilosis* -infected caterpillars was 60 hours. There was no significant difference between the survival curves of larvae infected with *C. parapsilosis sensu stricto* and *C* *. orthopsilosis* isolates.

## Discussion

In this study, we compared the pathogenic potential of *Candida* species belonging to the *psilosis* group based on the examination of the following features: (1) hydrolytic enzyme (protease and lipase) production, (2) pseudohyphae formation, (3) resistance to killing by macrophages, (4) macrophage-damaging capacity and (5) ability to cause lethal infection in *G* *. mellonella* larvae. As the production of hydrolytic enzymes has been associated with virulence for both *C. albicans* and *C. parapsilosis* [23,24], we first screened our strains for proteolytic and lypolytic activity. We found that almost all of the tested isolates of *C. parapsilosis sensu lato* group were positive for protease production, except for two *C* *. orthopsilosis* strains. While 81.0% of *C. parapsilosis sensu stricto* isolates were positive for lipase production, only 5.6% of *C* *. orthopsilosis* and none of the *C* *. metapsilosis* isolates possessed lypolytic activity. The pseudohyphae-producing ability also varied among *C. parapsilosis* and *C* *. orthopsilosis* strains, with 82.5% and 72.2% of the isolates forming pseudohyphae, respectively. However, all the examined isolates of *C* *. metapsilosis* were unable to develop pseudohyphae.

In order to compare the virulence of *C. parapsilosis sensu lato* species, we examined the interactions of murine and primary human macrophages with 9 different isolates each for *C. parapsilosis sensu stricto*, *C* *. orthopsilosis* and *C* *. metapsilosis* . Macrophages are phagocytic cells that play a crucial role in antifungal immunity. On the one hand, they are able to efficiently ingest and kill invading pathogens, which is especially important during early immune responses. On the other hand, they also play a role in the initiation of adaptive immune responses due to antigen presentation and secretion of different cytokines. Although the ability to avoid killing differed considerably between strains, *C. parapsilosis sensu stricto* isolates showed a significantly higher resistance to killing by primary human macrophages compared to *C* *. orthopsilosis* and *C* *. metapsilosis* isolates. However, the killing of *C. parapsilosis sensu stricto* isolates by J774 mouse macrophages did not differ significantly from that of *C* *. orthopsilosis* or *C* *. metapsilosis* strains. Although the reason for this interesting finding remains to be clarified, it suggests that the *in vitro* models for virulence studies must be chosen carefully, as the results may vary depending on how closely the system represents *in vivo* conditions. In our case, it is likely that either of the following accounts for the discrepancies: (1) cell lines do not express the same pattern recognition receptor repertoire as primary macrophages (e.g. mannose receptor is abundantly expressed in primary macrophages, but absent in J774 cells [25]), hindering the differentiation between species, or (2) the activation state of phagocytes is not the same resulting in different killing efficiency. However, when analyzing the macrophage-damaging capacity of *C. parapsilosis sensu lato* species, we obtained similar results with both J774 and primary human macrophages, showing that *C. parapsilosis sensu stricto* isolates are the most and *C* *. metapsilosis* strains the least cytotoxic. This finding is in agreement with the study of Sabino et al., which showed that *C. parapsilosis sensu stricto* isolates induced the highest and *C* *. metapsilosis* strains the lowest LDH release in J774 mouse macrophages [26]. In addition to fungal killing, we also analyzed the phagocytosis of the *psilosis* species by J774.2 macrophages using one representative isolate each of *C. parapsilosis sensu stricto*, *C* *. orthopsilosis* and *C* *. metapsilosis* . As assessed by quantitative imaging, J774.2 mouse macrophages showed higher phagocytic activity against *C. parapsilosis sensu stricto* and *C* *. orthopsilosis* compared to *C* *. metapsilosis* , which is in line with the findings of Orsi et al. who have shown that *C* *. metapsilosis* isolates are phagocytosed to a lower extent by microglial cells in comparison to *C. parapsilosis sensu stricto* or *C* *. orthopsilosis* [4]. It has been also shown by Orsi and colleagues that *C* *. metapsilosis* -containing phagosomes exhibit higher acidification degrees compared to those containing *C. parapsilosis sensu stricto* or *C* *. orthopsilosis* [4]. Although the examination of phagolysosome maturation is beyond the scope of our study, one may speculate that the increased acidification degree of phagosomes might be the reason for the finding that *C* *. metapsilosis* cells are killed with similar efficiency by J774.2 macrophages despite being phagocytosed to a lower extent compared to *C. parapsilosis sensu stricto* and *C. orthopsilosis*.We also analyzed the influence of lipase and pseudohyphae production on the virulence of *C. parapsilosis sensu lato* species. Our results show that the lipase and pseudohyphae positive isolates of the *psilosis* group had greater host-cell damaging capacity and were killed less efficiently by human macrophages, indicating that these factors provide a selective advantage to the *Candida* cells during infection.

To further investigate the pathogenic potential of the species of the *psilosis* group, we utilized an invertebrate model and examined the survival of *G* *. mellonella* larvae upon infection with different *C. parapsilosis* sensu lato isolates. The larvae of the greater wax moth, *G* *. mellonella* have been successfully used for several years as a new *in vivo* model for studying the virulence of various pathogenic bacteria and fungi [14,27]. Although there have been advances in *in vivo* challenge models, such as a recently described murine model of experimental vaginal candidiasis [7], we still lack an optimal vertebrate survival model to assess the pathogenicity of the *psilosis* species. Therefore, we decided to examine the survival of *G* *. mellonella* larvae after infection with different isolates of *C. parapsilosis sensu stricto*, *C* *. orthopsilosis* and *C* *. metapsilosis* . Overall, *C* *. metapsilosis* isolates were less virulent than *C* *. orthopsilosis* and *C. parapsilosis sensu stricto* strains. However, we found that there was a considerable variance in the pathogenicity of individual strains of the different *psilosis* species. This finding underscores the importance of isolate-specific investigations, especially because studies are shedding light on striking strain-dependent differences for both *C. albicans* and *C. parapsilosis* [26,28].

Overall, *C* *. metapsilosis* was the least virulent species of the *psilosis* group. Although there are several previous findings indicating that *C* *. metapsilosis* has decreased virulence compared to *C* *. orthopsilosis* or *C. parapsilosis sensu stricto* [3,4,26], our study is the most extensive to date that compares the virulence of the three species. Furthermore, this is the first study to use the invertebrate *G* *. mellonella* model to characterize the pathogenicity of *C. parapsilosis sensu lato* species.

## Supporting Information

Table S1 List of isolates used in the study. Extracellular enzyme activity and pseudohypha production of the isolates.
(XLS)
